# Supplementary material for: Launching of the Anaemia Research Peruvian Cohort (ARPEC): a multicentre birth cohort project to explore the iron adaptive homeostasis, infant growth and development in three Peruvian regions
Source: BMJ Open. 2021 May 13;11(5):e045609. doi: 10.1136/bmjopen-2020-045609 (PMC8126292; doi:10.1136/bmjopen-2020-045609)
Supplement: Author's manuscript [file bmjopen-2020-045609.draft_revisions.pdf]

BMJ Open is committed to open peer review. As part of this commitment we make the peer review history of every article we publish publicly available.

When an article is published we post the peer reviewers' comments and the authors' responses online. We also post the versions of the paper that were used during peer review. These are the versions that the peer review comments apply to.

The versions of the paper that follow are the versions that were submitted during the peer review process. They are not the versions of record or the final published versions. They should not be cited or distributed as the published version of this manuscript.

BMJ Open is an open access journal and the full, final, typeset and author-corrected version of record of the manuscript is available on our site with no access controls, subscription charges or pay-per-view fees (http://bmjopen.bmj.com).

If you have any questions on BMJ Open's open peer review process please email info.bmjopen@bmj.com

BMJ Open

Launching of the Anaemia Research PEruvian Cohort (ARPEC): A multicentre birth cohort project to explore the iron adaptive homeostasis, infant growth, and development in three Peruvian regions.

Journal:	*BMJ Open*
Manuscript ID	bmjopen-2020-045609
Article Type:	Protocol
Date Submitted by the Author:	06-Oct-2020

Complete List of Authors:	Montag, Doreen; Queen Mary University of London, Centre for Global Public Health<br>Delgado, Carlos; Instituto Nacional de Salud del Nino; Universidad Nacional Mayor de San Marcos, Pediatric, Facultad de Medicina<br>Quispe, Consuelo; Universidad Nacional de San Cristóbal de Huamanga<br>Wareham, David; Queen Mary University of London, Blizard Institute<br>Gallo, Valentina; University of Groningen, Campus Fryslan<br>Sanchez-Choy, Jose; Universidad Nacional Intercultural de la Amazonia<br>Sanchez, Victor; Instituto Nacional de Salud del Nino<br>Anaya, Ruth; Universidad Nacional de San Cristobal de Huamanga<br>Flores, Elaine; Queen Mary University of London, Centre for Global Public Health<br>Roca, Lorena<br>Mamani, Victor ; Instituto Nacional de Salud del Niño<br>Rivera Medina, Juan; Instituto Nacional de Salud del Niño; Universidad Nacional Mayor de San Marcos, Facultad de Medicina<br>Velasquez, Pablo; Instituto Nacional de Salud del Niño; Instituto Nacional Materno Perinatal<br>Del Aguila, Carlos; Instituto Nacional de Salud del Niño; Universidad Nacional Federico Villarreal<br>Prendergast, Andrew ; Queen Mary University of London,<br>Palomino, Julio; Universidad Nacional Santiago Antúnez de Mayolo

Keywords:	Anaemia < HAEMATOLOGY, Health policy < HEALTH SERVICES ADMINISTRATION & MANAGEMENT, MICROBIOLOGY, OBSTETRICS, PAEDIATRICS

SCHOLARONE™
Manuscripts

I, the Submitting Author has the right to grant and does grant on behalf of all authors of the Work (as defined in the below author licence), an exclusive licence and/or a non-exclusive licence for contributions from authors who are: i) UK Crown employees; ii) where BMJ has agreed a CC-BY licence shall apply, and/or iii) in accordance with the terms applicable for US Federal Government officers or employees acting as part of their official duties; on a worldwide, perpetual, irrevocable, royalty-free basis to BMJ Publishing Group Ltd ("BMJ") its licensees and where the relevant Journal is co-owned by BMJ to the co-owners of the Journal, to publish the Work in this journal and any other BMJ products and to exploit all rights, as set out in our licence.

The Submitting Author accepts and understands that any supply made under these terms is made by BMJ to the Submitting Author unless you are acting as an employee on behalf of your employer or a postgraduate student of an affiliated institution which is paying any applicable article publishing charge ("APC") for Open Access articles. Where the Submitting Author wishes to make the Work available on an Open Access basis (and intends to pay the relevant APC), the terms of reuse of such Open Access shall be governed by a Creative Commons licence – details of these licences and which Creative Commons licence will apply to this Work are set out in our licence referred to above.

Other than as permitted in any relevant BMJ Author's Self Archiving Policies, I confirm this Work has not been accepted for publication elsewhere, is not being considered for publication elsewhere and does not duplicate material already published. I confirm all authors consent to publication of this Work and authorise the granting of this licence.

For peer review only

Launching of the Anaemia Research PEruvian Cohort (ARPEC): A multicentre birth cohort project to explore the iron adaptive homeostasis, infant growth, and development in three Peruvian regions.

Doreen Montag[1,9x], Carlos A. Delgado[2,3x], Consuelo Quispe[4], David W Wareham[6], Valentina Gallo[1,6,7,8], Jose Sanchez-Choy[9], Victor Sanchez[2], Ruth Anaya[5], Elaine C. Flores [1,7,10], Lorena Roca[5], Victor Mamani[2], Juan Rivera Medina[2,3,11], Pablo Velasquez[2,12], Carlos Del Aguila[2,13], Andrew Prendergast[5], Julio Palomino[14]

[1] Institute of Population Health Science, Queen Mary University of London, London, UK
[2] Instituto Nacional de Salud del Niño, Lima, Peru
[3] Department of Paediatrics, Faculty of Medicine. Universidad Nacional Mayor de San Marcos. Lima, Peru
[4] Universidad Nacional de San Cristóbal de Huamanga, Ayacucho, Peru
[5]Blizard Institute, Queen Mary University of London, London, UK
[6]University of Groningen, Netherlands
[7]Department of Population Health, London School of Hygiene and Tropical Medicine, London, UK
[8] Department of Epidemiology and Biostatistics, School of Public Health, Imperial College London, London, UK
[9] Universidad Nacional Intercultural de la Amazonía, Pucallpa, Peru
[10]Universidad Católica los Ángeles de Chimbote, Instituto de Investigación, Chimbote, Peru
[11] Universidad Peruana Cayetano Heredia, Lima, Peru
[12]Instituto Nacional Materno Perinatal, Lima, Peru
[13]Universidad Nacional Federico Villarreal, Lima, Peru
[14]Universidad Nacional Santiago de Antuñez de Mayolo, Huaraz, Peru

[x] Equal contribution

Word count: Abstract: 317 Main Text: 5253 Figures: 2

Keywords: Anaemia, iron deficiency, Ecohealth, cohort, infant, Peru

Corresponding author
Dr Doreen Montag
Senior Lecturer of Global Health
Institute of Population Health Science, Queen Mary University of London, London, UK
Yvonne Carter Building, 58 Turner Street, London, E1 2AB
d.montag@qmul.ac.uk

For peer review only

Abstract

Background: Preventing infantile anaemia and ensuring optimal growth and development during early childhood, particularly in resource-constrained settings, represent an ongoing public health challenge. Current responses are aligned to treatment-based solutions, instead of determining the roles of its interrelated causes. This project aims to assess and understand the complex interplay of eco-bio-social-political factors that determine infantile anaemia to inform policy, research design and prevention practices.

Methods: This is a longitudinal birth cohort study including four components: 1. Biological, will assess known blood markers of iron homeostasis and anaemia and stool microbiota to identify and genetically analyse the participants' flora; 2. Ecological, will assess and map pollutants in air, water and soil and evaluate features of nutrition and perceived food security; 3. Social, which will use different qualitative research methodologies to explore key stakeholders and informants' perceptions related to nutritional, environmental and anaemia topics, participant observations and a participatory approach; and 4. A Political analysis, to identify and assess the impact of policies, guidelines and programs at all levels for infantile anaemia in the three regions. Finally, we will also explore the role of social determinants and demographic variables longitudinally for all study participants. This project aims to contribute to the evidence of the interrelated causal factors of infantile anaemia, addressing the complexity of influencing factors from diverse methodological angles. We will assess infantile anaemia in three natural regions of Peru, including newborns and their mothers as participants, from childbirth until their first year of age.

Ethics and Dissemination: Ethical approval was obtained from the Institutional Research Ethics Committee of the Instituto Nacional de Salud del Niño (Lima, Peru), CIEI-043-2019. It has also been granted by the Ethical Committee of Queen Mary University of London (London, United Kingdom). Dissemination across stakeholders is taking part as a continues part of the research process.

Summary

'Strengths and limitations of this study'

- There is scarce empirical evidence on the interrelated causes of infantile anaemia in LMICs settings. This project will address the complex interplay between the eco-bio-social-political factors that determine infantile anaemia.
- The project outcomes will inform policy, research design and prevention practices from a multidisciplinary evidence-based perspective.

For peer review only

Introduction

Iron deficiency (ID) anaemia (IDA) is a pressing global health challenge, which impacts the auditory, visual, and neurological development of children[1-3]. By 2016, the worldwide prevalence of anaemia in children <5 years, reported in the WHO Global Health Observatory data repository was 41.7 % [38.1-45.9]. The major risk factors for IDA are food insecurity[4], malnutrition and poverty, all of which will be exacerbated under current and future climate conditions[5]. The Anthropocene is marked by global change impacting planetary processes, including biodiversity and land-use changes[6]. Characterising and solving the underlying complexity of the factors that interact and determine IDA for millions of children, living in different circumstances globally, will be crucial to adapt to future risks and to mitigate the burden of disease by developing a durable environmental governance focused on health[7-9].

In Peru, as in other low-and-middle-income countries (LMICs), ID and IDA represent a high burden of disease. Between the years 2000 and 2010, the prevalence was over 50% in children aged 6 to 35 months and by 2017 it remained around 40%[10]. Nationally in the same period, anaemia prevalence decreased by about 10%[11]. However, from 2012 to date, the prevalence rates of anaemia remain around 42.2%[10 12].

It is important to highlight, though, that treating a deficiency does not necessarily mean supplementing it. During early life, iron homeostasis controls are absent or limited, because the regulation mechanisms for this process develop in later infancy[13]. Prescription of iron supplementation during the first months of life, whether during breastfeeding or mixed breastfeeding, may interfere with nutrient absorption and may adversely affect the intestinal microbiome[14 15]. Also, there is sparse evidence that iron supplementation may correct neurodevelopmental deficits caused by a lack of iron in early childhood[16].

The eco-bio-socio-political framework in Peru

Political & economic factors

Over the last decades there has been a steady increase of the Gross domestic product (GDP) in Peru[17], with up to over 8% per year growth between the years 2000 and 2018[17] reflecting a commodity boom. The GDP, as an indicator of macro-economic development, should have had a direct impact on poverty reduction, decreased food insecurity indicators and improved health outcomes, leading to reduced IDA prevalence. However, this has not happened: high rates of infant anaemia still exist[18] suggesting a lack

For peer review only

of a continuous systemic approach by the government, despite its importance on social and economic indicators for the Peruvian society.

Social factors

Food insecurity in Peru is rooted in Peruvian colonial history and imbalanced power relations. It is distributed unevenly in each ecological region, often merely addressed as a problem of poverty, which is poorly overseen by policies and interventions over the last 70 years[19]. Food insecurity and malnutrition are consequences of multidimensional factors related to broader social and economic inequalities. These, in turn, influence rural-urban migration patterns and configures budgetary restrictions for food and nutrition of women in their homes[20 21]. Gender relations within households' further influence power dynamics and subsequently has a direct impact on infant nutrition[22].

Biological factors

Biological factors encompass microbiota development and iron homeostasis in breastfeeding infants. During breastfeeding, there is a predominance of *Bifidobacterium* bacteria, that represent 60-90% of the total microbiota, due to the rich supply of oligosaccharides in breast milk. As complementary food gets introduced, these are successively displaced by *Firmicutes* and *Bacteroidetes*, capable of absorbing complex polysaccharides derived from plant products appropriate for the stage of infant feeding and growth. Given the large concentration of lactoferrin (an iron-transporting protein) in breast milk, and its resistance to proteolysis, it can be detected in breastfeeding stools[23]. Optimal microbiota succession can be disrupted by environmental factors, such as exposure to unclean water and lack of sanitation, early introduction of complementary feeding during the period of exclusive breastfeeding, and the quality of the infant diet.

Ecological & environmental factors

Biodiversity loss, increased urbanisation and deteriorating ecosystems have a documented impact on gut microbiota and health[24 25]. Rook and Knight[26] argue that biodiversity of the soil, such as the green spaces that children mature in, plays a vital role in the bacteria induced immune modulation, and subsequently influences microbiota development and homeostasis. Anthropogenic climate change is altering biodiversity and iron and other mineral content of certain foods, further restricting access to food across vulnerable population groups and increasing food insecurity[27 28].

For peer review only

Water quality is influenced by biodiversity, deforestation, mining activities and general pollution. Water pollution, specifically, can lead to increased parasitic infections in infants, which in turn can compete for iron in the intestine[24 29]. Environmental enteropathy (EE), also called environmental enteric dysfunction[30], is a subclinical disorder of the small intestine which alters the structure and function of the gut among children living in settings with environmental contamination and high poverty rates. EE reduced gut barrier function, and intestinal malabsorption and all of these processes may contribute to IDA[31 32].

Rationale of the project

Given the complexity of the factors potentially increasing the risk of IDA, a comprehensive approach is needed to investigate and disentangle such complexity. This entails examining in detail the ecological, biological, social and political factors that would increase the risk for developing IDA in the first year of life among 330 children in Lima (Coast), Ayacucho (Andes) and Pucallpa (Amazon), who will be born to healthy mothers without anaemia during the year 2021. We are proposing an eco-bio-socio-political framework, which is influenced by the work edited by Charron[33] on 'Ecohealth research in practice', that emphasises the importance of participation of all stakeholders and an interdisciplinary methodology, gender equity and sustainability to increase potentially lasting policy impacts. With the base of an initial simple diagram (Figure 1) and the research outcomes, we plan to approach and comprehend in-depth the complex interplay between the selected factors which will allow us to further develop this framework.

Figure 1. The Eco-bio-socio-political framework applied to iron deficiency anaemia in children in Peru - Source: Own elaboration (DM)

For peer review only

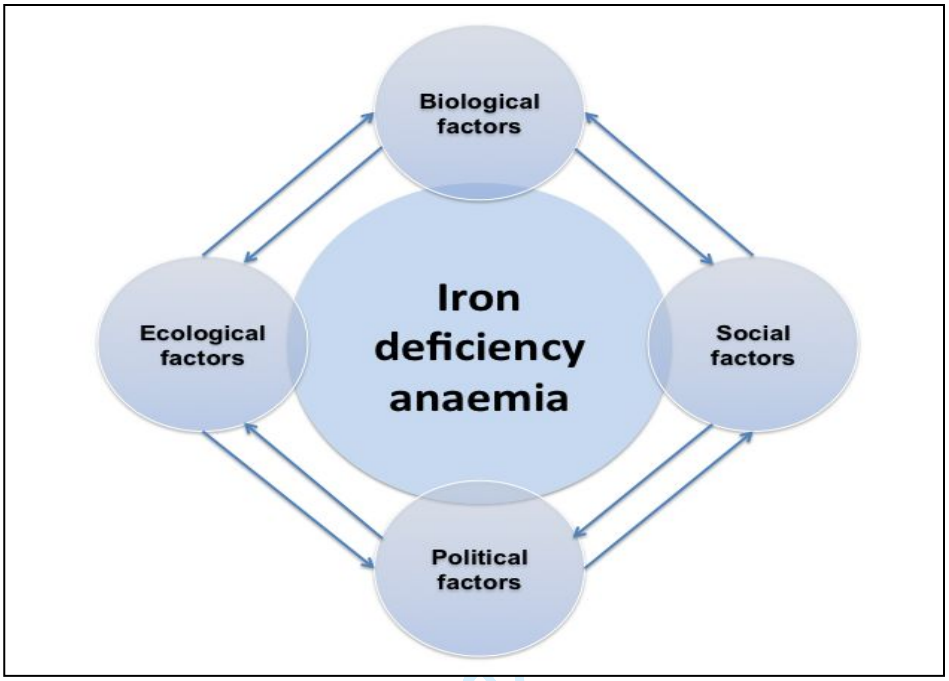

The Anemia Research Peruvian Cohort (ARPEC) project seeks to diminish the scientific and technical evidence-base gap, supporting the development of comprehensive and sustainable policy[7 20 21 34]. In Peru, as in most of Latin America, the majority of anaemia is related to ID, due to lower levels of iron in the diet and its coexistence with high rates of gastrointestinal issues that impairs iron absorption[35-37]. For example, in the Andean region of Ayacucho, children's dietary iron consumption is almost 25% below the recommended iron requirements by age[38]. Despite this, the interdisciplinary empirical literature on the tangled causes and long-term determinants of this highly prevalent challenge is almost non-existent.

The ARPEC project status is updated regularly on its webpage https://www.qmul.ac.uk/arpecproject/

Aim and objectives of the study

Understand the complexity of the eco-bio-socio-political factors that influence food insecurity of breastfeeding mothers and their new-borns in three Peruvian regions, altering mechanisms of adaptive iron homeostasis in infants within the Anthropocene.

Objectives

1. To evaluate the effects of infant exposure to ecological / environmental factors, including nutrition, water quality and air and soil pollution, and their potential influence on gut microbiome development and the mechanisms of adaptive iron homeostasis in infants.

2. To assess the contribution of biological factors, such as succession of the intestinal microbiota, to the incidence of IDA in breastfed infants during the first year of life. We will focus especially on those organisms with siderophore genes and a competitive role in intestinal iron absorption.
3. To identify social factors influencing dietary patterns and food insecurity and their association with ID or IDA in breastfed babies during the first year of life.
4. To analyse political factors, including success or failure of previous policies and lasting and sustainable policies, in addressing food insecurity and IDA in different Peruvian regions.
5. To develop a framework able to explain the complex interaction and interdependence of the eco-bio-social-political factors determining IDA in Peru.

Methods

Study design

ARPEC project is a prospective observational multi-centre birth cohort study that will be conducted in three regions within Peru: the coast (Lima), the Andes (Ayacucho) and the Amazon rainforest (Pucallpa) with integrated quantitative and qualitative methodologies.

Setting

Peru is the third most megadiverse country in the world, home to 55 indigenous groups (51 in the Amazon and 4 in the Andes) with 48 different indigenous languages[39]. Diversity is reflected in the nutritional and food practices throughout the country. This study involves inhabitants from three different regions: 1. Lima, as the capital of Peru, is located at 101 meters above sea level in the central coast. Lima encompasses almost 9.5 million inhabitants (32.3% of the national population) in a desert environment. It has the lowest proportion nationally of children >5 years old (5.1%). By 2018, in the urban Lima, the prevalence rates of anaemia in children between 6-35 months was 35.9%[12] and in 2017, among women between 15 and 49 years old was 23.1%[10]. 2. Pucallpa, the capital of the regional department of Ucayali, located at 154 meters above sea level, in the central Peruvian Amazon rainforest. It has almost half a million inhabitants (1.7% of the national population). Stunting among children <5 was 24.8%, one of the highest in the country. The prevalence of anaemia in children between 6-35 months was 56.4% in 2018[12], and the highest prevalence was observed in children between 0-8 months (62%)[11]. 3. Ayacucho, is located at 2,760 meters above sea level in the Andes of South-eastern Peru. This region was the most affected during the bloody internal conflict in Peruvian recent history (1980-2000). The region has 616,176 inhabitants (2.1 % of the national population). In 2018, stunting

For peer review only

prevalence was 18.5% in children <5 years of age, and anaemia affected 49.3% of children aged 6-35 months[12]. Ayacucho women of childbearing age constitute 17% of the total population (21% nationwide) and 31.5% of pregnant women[10].

Participant recruitment and selection criteria

The project will identify, select and recruit 330 pairs of mothers and new-borns who will be involved in the study. Potentially eligible participants will be identified from the official registries of full-term pregnancies at the recruitment centres in the three cities. We will consider the following inclusion criteria: singleton births, delivered vaginally, with umbilical cord clamping after ≥60 seconds, new-borns weighing >2,500 grams, mother-baby hospitalised jointly and discharged before 72 hours of life, without diagnosis of perinatal infection, birth asphyxia or congenital malformation. Participant mothers aged +18 years, who are capable of understanding study procedures, providing informed consent, and full-time residents in the study area. The mothers will be assessed during the month before the childbirth, at the last prenatal check-up or retrospectively through review of clinical records. Exclusion criteria are: mothers with a diagnosis of anaemia on the last prenatal haemoglobin levels; who had delivery via caesarean section; received antibiotics during the last month of pregnancy and the first week of life (mothers or their new-borns).

Sample size and power calculation

The study sampling will be purposeful. We plan to have a total sample of 330 mother/child pairs, recruiting 110 new-borns from each region, totalling 660 participants, and assuming a maximum potential loss of follow-up of 10%. Our hypothesis is that selected ecological, biological, social, and political factors increase the risk of IDA in the first year of life among the new-born's participants from the three regions. To test this association, we will have a power of 90% at a significance level of 5%, to detect an average difference of 0.5 g/dL between two subgroups, and 75% power to detect an average difference of 0.4 g/dL. Both are important differences for public health decisions and are associated with relevant outcomes such as early childhood development. We estimate that the level of haemoglobin in infants will be similar to that of their mothers, as has been reported for the city of Pucallpa in Peru[40] (average 11.5 g/dL, DS 1.3).

For peer review only

Study procedures

Current knowledge review

We will perform an in-depth review of the literature, combining a systematic search and review with a policy analysis. The systematic review will focus on current knowledge on ID and IDA in Peru and the impact of environmental factors on IDA. The policy analysis will be focused on stakeholders, content, context, and capacity analysis to understand past successes and the underlying reasons on why, where and what has not been successful tackling IDA in Peru[41 42]. Findings from reviews will inform the qualitative research component planning and methodology, comprising in-depth interviews (IDIs) and focus groups discussion (FGDs) sessions.

Assessments of anaemia

We will assess IDA through haemoglobin (Hb), ferritin and C-reactive protein (CRP) levels in blood samples at baseline (birth) and during follow-up study visits at 4, 8, and 12 months of age[43]. Mothers will be examined after childbirth and before hospital discharge. In addition, concentrations of serum hepcidin, soluble transferrin receptor (sTFR) and lead will also be evaluated. We will use WHO criteria to adjust ferritin values for inflammation[44], as reflected by the CRP[45]. We will follow standard guidelines to draw laboratory samples from children, accompanied by their mothers or primary caregivers.

Basic anthropometric measurements

We will measure weight and length of children at baseline and at each follow-up visit, with a digital calibrated scale and an infant meter, respectively, following standard local procedures. Additionally, we will measure head circumference and abdominal circumference with a non-elastic metric tape. Weight, height and abdominal circumference of mothers will be taken at baseline and at the 12-month visit, following standard assessment procedures for adults. All measurements will be performed by trained fieldworkers, with experience in local health services. Child development will be assessed using a locally validated version of the Child Welfare Survey (SWYC)[46]. The SWYC is a free screening instrument and requires almost 15 minutes to complete. It includes questionnaires adapted to age milestones to assess three domains of child functioning: Developmental Domain, Emotional/Behavioral Domain and Family Context.

Metagenomic analysis of the microbiota

The child's stool microbiota will be analysed using whole genome sequencing techniques during the three follow-up visits at 4, 8 and 12 months of life. The samples will be collected, processed, stored, and

For peer review only

shipped for analysis from Peru to the Blizard Institute, Queen Mary University of London (QMUL). We will also undertake phenotypic analysis, using selective culture media, to isolate viable bacterial pathogens in a central laboratory in Lima. Selective chromogenic bacterial culture media will be used for primary isolation and quantification of *Enterobacterales, Enterococci, Bifidobacterium, Lactobacilli, Bacteroides, Clostridia, Candida spp* and siderophore-producing isolates (CAS agar). Molecular analysis and next generation sequencing (NGS) of the faecal microbiome will be performed by metagenomic shotgun sequencing[47 48]. The DNA of the entire bacterial community will be extracted, fragmented and sequenced using Illumina HiSeq technology. Bioinformatic analysis of sequence data will be performed using the CosmosID Metagenomics Cloud application (https;//app.cosmosid/login) (Rockville, MA) to enable taxonomic profiling of bacteria, viruses, phages, fungi, protists, microbial resistome and virulome.

Water quality analysis

Sampling for water quality analysis will be conducted in two different seasons (dry and wet) and within comparative geographical reference points in each region (Pucallpa, Ayacucho, Lima), considering their relationship to other human activities, including mining, lack of sewage and other sanitary installations that have an impact on water quality. We will determine during fieldwork collection the pH, conductivity, turbidity, and percentage of dissolved oxygen in water samples. In addition, we will assess under laboratory conditions the levels of metals (such as iron, manganese, aluminium, copper, zinc, lead, mercury), hardness characteristics, presence of nutrients (such as phosphate, nitrate), and microbiologic characteristics (such as the presence and quantity of heterotrophic bacteria, total coliforms and thermotolerant coliforms) following the government's permitted parameters[49]. The assessments will follow the protocols of Standard Methods for Water and Wastewater[50] and laboratory guidelines of the World Health Organization.

Air quality analysis

We will evaluate air quality across the three regions using data from different monitors, satellite reports and field collection at pre-identified geographical landmarks during the study visits. Environmental exposure to air pollutants, fine particles equal to or less than 2.5 microns in diameter (PM2.5) and NO_2 may produce systemic inflammation and determine changes in the bone marrow and act as a direct risk factor for the development of anaemia[51 52]. A recent study conducted in Lima, Peru, determined a significant association between the increase in the level of PM 2.5 measured in the environment, with

For peer review only

decreased haemoglobin values and an increase in the prevalence of moderate and severe anaemia in children under 5 years[53].

Geographic mapping

We will also explore the proposed relationship between environmental exposures and microbiota characteristics using the GIS georeferencing system. This will allow us to map the prevalence of anaemia prevalence through estimating the approximate distance to the child's home, the specific microbiota differences and the biodiversity level of the green areas surrounding the households[26].

Sociodemographic and nutritional evaluation procedures

We will assess socioeconomic and food practices variables among all participants. The nutritional instrument will be locally piloted and adapted for each region to provide information on the food groups consumed by the mother and infant beyond 4 months of age when new food is being introduced. This will vary within regions, by seasonality factors and food access. We will also use locally pilot visual materials to ease the description and characteristics of foods, drinks, and portion sizes of the food commonly consumed by the participants.

We acknowledge the potential importance of the participant's various socio-cultural backgrounds (Shipibo-Konibo, Quechua) and the different feeding practices during weaning, which will be explored in-depth through qualitative methodology. Our approach aims to be participatory since inception, following Eco health approaches[33] and has a high potential to develop participatory and lasting prevention policies. We will base our analysis on the known nutritional content of fruits and legumes nationally[54].

Qualitative Research Procedures

We will conduct in-depth interviews with participating mothers, regional and national key informants, and local stakeholders. We will aim to interview between 25-30 mothers (8-10 from each region) to reach saturation, selected by convenience. For the stakeholder interviews, one key stakeholder per institution will be selected. Given the high fluctuance of stakeholders in institutions and to comprehend the challenges of anaemia policies from a historic perspective a snowball method will be used to determine stakeholders and provide a comprehensive insight[55]. The purpose of interviews among

For peer review only

stakeholders is to evaluate their perceptions of what are the causes for IDA and their opinion on the historical success or failure of policy interventions related to anaemia. 3-6 FGDs[56] will be conducted among mothers and stakeholders, separately, in each location to identify, discover and review important themes and provide meaning[57]. FGDs among key stakeholders in each region and on a national level will address two main areas: First, the perceived relationship between nutritional and environmental factors and the potential risks for the development or worsening of the iron deficiency anaemia among children. Secondly, they will address the policy level, which will be divided into two phases: 1. The experiences and perceptions of effective past and current policies and barriers to effective implementation of governmental interventions and lasting policies and 2. During the last part of the research period, after the presentation of the results, to discuss and inform potential policy implementation based on the research results and practical aspects of transforming policies into practice. Finally, we anticipate conducting a public engagement and participatory activity with a group of participating mothers from each region. This will use photography as a means of non-verbal expression, allowing participants to identify, select, register and present everything that they consider important and related to the health of their new-borns and the risks related to the development of anaemia, following the PhotoVoice © method[58].

Operationalization of the variables

The list of variables and their characteristics will be included in the study protocol, instruments, manuals, and standard operations procedures.

Data analysis

We will conduct biological (microbiota/stool, water, blood analysis), socio-cultural (nutrition, environment, policy), geographic-mapping (GIS-location relationship between green spaces, biodiversity and gut microbiota), and mixed-methods research (nutrition/household survey, qualitative methodology).

Quantitative data analysis

We will review the distribution of any missing values and evaluating imputation options, normality for all quantitative variables and their correlations. We will conduct descriptive analysis of the distribution of all microbiome and lifestyle data between the different haemoglobin levels across all participants.

Surveyed quantitative variables will be categorised appropriately for each follow-up visit and we will estimate their changes over time. Biological samples will be analysed to evaluate its microbiota content, and we will identify the most frequent taxa for each sample. Repeated measurements will allow us to

For peer review only

calculate the largest changes in microbiota composition during follow-up period. Principal Component Analysis will be used to identify the most distinctive taxa and their associated patterns of changes with the lowest haemoglobin levels at the end of the study period. These in turn will be analysed in association with other lifestyle variables to identify possible causal pathways.

A multivariate logistic regression model will be used to investigate the cross-sectional association between potential risk factors and haemoglobin levels in children, in each visit. Additionally, information on selected exposures will be collected four times to calculate cumulative exposure, which will be then evaluated against ID and IDA at the end of study period. Both cumulative exposures and their changes will be included in a final Cox proportional hazards model, including exposure variables at the starting point, and their relevant changes, to explore the risk associated with lower haemoglobin levels, during the first year of life.

Qualitative analysis

Data obtained from participant observations, focus group sessions and in-depth interviews will be audio-recorded and transcribed *verbatim*. This will later be segmented, entered, coded, and analysed to identify a list of relevant themes and key concepts related to the topics of interest. Coding will continue throughout the research process, using the grounded theory approach to data analysis, and informing follow-up interviews and focus groups[59]. The analysis will compare the information by different subgroups of participants to identify and describe the similarities and divergences between key stakeholders, participants from different regions, age groups, and those living across different environmental and nutritional risks. The analysis will also include field notes, direct observation, and minutes of the researchers' meetings.

Ethics

Ethical approval was obtained from the Institutional Research Ethics Committee of the Instituto Nacional de Salud del Niño (Lima, Peru), CIEI-043-2019. It has also been granted by the Ethical Committee of Queen Mary University of London (London, United Kingdom). Further to that, institutional support was sought from local authorities, particularly indigenous government organisations, local hospitals and health centres.

Written informed consent will be sought from all participants prior to enrolment. Oral consent and permanent explanation of research purposes will continue throughout the study.

For peer review only

To protect participant's confidentiality, all collected data will be linked to a unique identification code. Data will be recorded in paper form and electronically. The electronic information will be stored, backed-up, and secured by password protection in the RedCap© server. Paper forms will be stored in secure locked cabinets at the three Peruvian sites. All confidential information, including participant's contact details will also be stored in secured locations, accessible only to authorized research staff. Long-term data management will comply QMUL's research policy.

Any requests for biological samples will be processed following international standards. Importantly, only processed stool samples will be sent to the UK.

For peer review only

Plan for results dissemination

The study team has been following and will adhere to a detailed plan for dissemination of study outcomes, that will be used to publicise the research at a national and regional level. The outline dissemination methods will include periodic meetings with stakeholders, academic institutions that directly and indirectly collaborate with the study team, scientific publications in peer-reviewed journals, presentation in scientific events to allow engagement with the wider international academic society in the discussion of research results, creation and release of integral policy briefs for stakeholders at the regional and national level in Peru, teaching through research-based teachings in relevant participating institutions and modules and presence in social media and a dedicated study website and logo (figure 2).

Author contribution

Doreen Montag – Principal investigator (MRC) at QMUL, London. Conceived study ideas, drafted protocol, and overall responsibility of study design and implementation. Drafted the current manuscript

Carlos A. Delgado – Director of the Research Circle (FONDECYT) Principal Investigator at INSN, Lima. Conceived study ideas, major contribution to protocol, and overall responsibility of study design and implementation in Peru.

Consuelo Quispe – Local Principal Investigador for UNSCH, Ayacucho. Responsible of conceiving and operationalising anthropometric data collection.

David Wareham – Co-investigator at QMUL. Responsible for conceiving and operationalising genetic analyses of the microbiome. Theoretical and logistic input on faeces sample collection and microbiota analyses

Jose Sanchez Choy – Co-investigator at UNIA. Manager of data collection in Pucallpa

Victor Sanchez – Co-investigator at INSN. Overall input on study design and biomedical background and measurements

Ruth Anaya – Co-investigador, UNSCH, Ayacucho. General input on operationalising anthropometric data collection.

Valentina Gallo – Co-investigator at QMUL, London. Overall advice on epidemiological study design and statistical analyses. General input on biomedical background and measurements for neurodevelopmental issues

Andrew Prendergast – Co-investigator at QMUL, London. Overall input on study design and implementation

Lorena Quispe Roca -Research Assistant at UNSCH, Ayacucho, general input on anthropometric data collection and study context in Ayacucho.

Elaine C. Flores – Study -coordinator, coordination of protocol writing, collaborating in this manuscript drafting.

Victor Mamani- Nutrition expert, intellectual contribution to nutritional component.

Juan Rivera Medina-Co-investigator at INSN, intellectual contribution to biological research component.

Pablo Velásquez, Co-investigator at INSN, intellectual contribution to biological research component.

Carlos Del Aguila- Co-investigator at INSN, intellectual contribution to biological research component.

Julio Palomino – Local principal investigator for UNIA, Pucallpa. Responsible of conceiving and operationalising environmental data collection. Theoretical and logistic input on faeces sample collection, microbiota, and environmental analyses.

Funding

This project is supported by the Medical Research Council – MRC grant number MR/S024654/1 and by the Fondo Nacional de Desarrollo Científico, Tecnológico y de Innovación Tecnológica – FONDECYT, grant number 033-2019. AJP is funded by the Wellcome Trust (108065/Z/15/Z).

Competing interest statement

None of the authors declare any competing interest

For peer review only - http://bmjopen.bmj.com/site/about/guidelines.xhtml

Figures

Figure 2

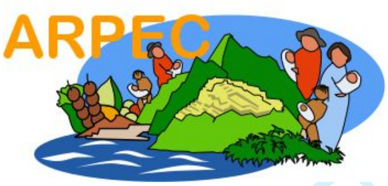

Arpec logo by Daddo Illustrations at www.daddoillustrations.com.

For peer review only

References

1. Algarín C, Peirano P, Garrido M, et al. Iron deficiency anemia in infancy: long-lasting effects on auditory and visual system functioning. *Pediatric research* 2003;53(2):217.
2. Allali S, Brousse V, Sacri AS, et al. Anemia in children: prevalence, causes, diagnostic work-up, and long-term consequences. *Expert Rev Hematol* 2017;10(11):1023-28. doi: 10.1080/17474086.2017.1354696 [published Online First: 2017/10/13]
3. Lozoff B, Beard J, Connor J, et al. Long-lasting neural and behavioral effects of iron deficiency in infancy. *Nutr Rev* 2006;64(5 Pt 2):S34-43. [published Online First: 2006/06/15]
4. FAO. State of Food Insecurity in the World: Meeting the 2015 International Hunger Targets: Taking Stock of uneven Progress. Rom: FAO, 2015.
5. Crutzen PJ. Geology of Mankind. In: Crutzen PJ, Brauch HG, eds. Paul J Crutzen: A Pioneer on Atmospheric Chemistry and Climate Change in the Anthropocene: Springer 2016:211-15.
6. Rockström J, Steffen W, Noone K, et al. Planetary Boundaries: Exploring the Safe Operating Space for Humanity. *Ecology and Society* 2009;14(2):32.
7. Montag D, Kuch U, Rodriguez L, et al. Overview of the Panel on Biodiversity and Health under Climate Change In: Diversity CoB, ed. The Lima Declaration on Biodiversity

Climate Change: Contributions from Science to Policy for Sustainable Development: Convention of Biological Diversity 2017:91-108.
8. Watts N, Adger WN, Agnolucci P, et al. Health and Climate Change: Policy Responses to Protect Public Health. *The Lancet* 2015;386(10006):1861-914.
9. Whitmee S, Haines A, Beyrer C, et al. Safeguarding Human Health in the Anthropocene Epoch: Report of the Rockefeller Foundation–Lancet Commission on Planetary Health. *The Lancet* 2015;386(10007):1973-2028. doi: 10.1016/S0140-6736(15)60901-1
10. INEI. Instituto Nacional de Estadística e Informática. Encuesta Demográfica y de Salud Familiar - ENDES 2017. Lima: Instituto Nacional de Estadística e Informática, 2017.
11. INEI. Instituto Nacional de Estadística e Informática. Encuesta Demográfica y de Salud Familiar - ENDES 2016. Lima: Instituto Nacional de Estadística e Informática, 2016.
12. INEI. Instituto Nacional de Estadística e Informática. Encuesta Demográfica y de Salud Familiar - ENDES 2019 (resultados preliminares). Lima: Instituto Nacional de Estadística e Informática, 2019.
13. Lonnerdal B. Development of iron homeostasis in infants and young children. *Am J Clin Nutr* 2017;106(Suppl 6):1575s-80s. doi: 10.3945/ajcn.117.155820 [published Online First: 2017/10/27]
14. Dewey KG, Domellöf M, Cohen RJ, et al. Iron supplementation affects growth and morbidity of breast-fed infants: results of a randomized trial in Sweden and Honduras. *The Journal of nutrition* 2002;132(11):3249-55.
15. Paganini D, Zimmermann MB. The effects of iron fortification and supplementation on the gut microbiome and diarrhea in infants and children: a review. *Am J Clin Nutr* 2017;106(Suppl 6):1688s-93s. doi: 10.3945/ajcn.117.156067 [published Online First: 2017/10/27]
16. Cerami C. Iron Nutriture of the Fetus, Neonate, Infant, and Child. *Ann Nutr Metab* 2017;71 Suppl 3:8-14. doi: 10.1159/000481447 [published Online First: 2017/12/22]
17. The World Bank Group. Peru 2019 [cited 2019. Available from: https://data.worldbank.org/country/peru accessed February 27th, 2019 2019.
18. Alcázar L. The economic impact of anaemia in Peru: Lima 2013.
19. Smith LC, El Obeid AE, Jensen HH. The geography and causes of food insecurity in developing countries. *Agricultural economics* 2000;22(2):199-215.
20. Leatherman T, Jernigan K. The Reproduction of Poverty and Poor Health in the Production of Health Disparities in Southern Peru. *Annals of Anthropological Practice* 2014;38(2):284-99.
21. Oths KS, Smith HN, Stein MJ, et al. A decade of rapid change: Biocultural influences on child growth in highland Peru. *American Journal of Human Biology* 2018;30(2):e23072.
22. Serrano CA, Harris PR. [Development of intestinal microbiome in children. Impact on health and disease]. *Rev Chil Pediatr* 2016;87(3):151-3. doi: 10.1016/j.rchipe.2016.04.002 [published Online First: 2016/05/12]
23. Lonnerdal B, Georgieff MK, Hernell O. Developmental physiology of iron absorption, homeostasis, and metabolism in the healthy term infant. *The Journal of Pediatrics* 2015;167(4):S8-S14.
24. WHO/CBD. Connecting Global Priorities: Biodiversity and Human Health. A State of Knowledge Review.: World Health Organization and Secretariat of the Convention on Biological Diversity 2015.

For peer review only

25. Rook GA. Regulation of the Immune System by Biodiversity from the Natural Environment: An Ecosystem Service Essential to Health. *Proceedings of the National Academy of Sciences* 2013;110(46):18360-67.
26. Rook GA, Knight R. Environmental Microbial Diversity and Noncommunicable Diseases. In: WHO/CBD, ed. Connecting Global Priorities: Biodiversity and Human Health A State of Knowledge Review. Geneva: World Health Organization and Secretariat of the Convention on Biological Diversity 2015:151-64.
27. Torp Austvoll C, Gallo V, Montag D. Health impact of the Anthropocene: the complex relationship between gut microbiota, epigenetics, and human health, using obesity as an example. *Glob Health Epidemiol Genom* 2020;5:e2. doi: 10.1017/gheg.2020.2 [published Online First: 2020/05/05]
28. Phalkey RK, Aranda-Jan C, Marx S, et al. Systematic review of current efforts to quantify the impacts of climate change on undernutrition. *Proceedings of the National Academy of Sciences* 2015;112(33):E4522-E29.
29. Hudak L, Jaraisy A, Haj S, et al. An updated systematic review and meta-analysis on the association between Helicobacter pylori infection and iron deficiency anemia. *Helicobacter* 2017;22(1) doi: 10.1111/hel.12330 [published Online First: 2016/07/14]
30. Weinhouse C, Ortiz EJ, Berky AJ, et al. Hair Mercury Level is Associated with Anemia and Micronutrient Status in Children Living Near Artisanal and Small-Scale Gold Mining in the Peruvian Amazon. *Am J Trop Med Hyg* 2017;97(6):1886-97. doi: 10.4269/ajtmh.17-0269 [published Online First: 2017/10/11]
31. Owino V, Ahmed T, Freemark M, et al. Environmental Enteric Dysfunction and Growth Failure/Stunting in Global Child Health. *Pediatrics* 2016;138(6):e20160641. doi: 10.1542/peds.2016-0641 [published Online First: 2016/12/13]
32. Watanabe K, Petri WA, Jr. Environmental Enteropathy: Elusive but Significant Subclinical Abnormalities in Developing Countries. *EBioMedicine* 2016;10:25-32. doi: 10.1016/j.ebiom.2016.07.030 [published Online First: 2016/08/09]
33. Charron DF. Ecohealth Research in Practice. Ottawa, Cairo, Dakar, Montevideo, Nairobi, New Delhi, Singapore: Springer, IDRC, CRDI 2012.
34. Acosta AM, Haddad L. The politics of success in the fight against malnutrition in Peru. *Food Policy* 2014;44:26-35.
35. Mujica-Coopman MF, Brito A, Lopez de Romana D, et al. Prevalence of Anemia in Latin America and the Caribbean. *Food Nutr Bull* 2015;36(2 Suppl):S119-28. doi: 10.1177/0379572115585775 [published Online First: 2015/07/01]
36. Prendergast A, Kelly P. Enteropathies in the developing world: neglected effects on global health. *Am J Trop Med Hyg* 2012;86(5):756-63. doi: 10.4269/ajtmh.2012.11-0743 [published Online First: 2012/05/05]
37. Bartelt LA, Bolick DT, Guerrant RL. Disentangling Microbial Mediators of Malnutrition: Modeling Environmental Enteric Dysfunction. *Cell Mol Gastroenterol Hepatol* 2019;7(3):692-707. doi: 10.1016/j.jcmgh.2018.12.006 [published Online First: 2019/01/11]
38. Fundación Accion Contra el Hambre. Aproximación al Consumo de Alimentos y Prácticas de Alimentación y Cuidado Infantil en Niños y Niñas de 6 a 23 meses de Edad - Realizado en familias de la provincia de Vilcashuamán, región Ayacucho. . In: Rivera HTF, ed. 1 ed. Lima: Acción contra el hambre

, 2012:1-74.
39. INEI. Instituto Nacional de Estadística e Informática 2009 [
40. Gonzales GF, Tapia V, Gasco M, et al. [Maternal hemoglobin in Peru: regional differences and its association with adverse perinatal outcomes]. *Rev Peru Med Exp Salud Publica* 2011;28(3):484-91. [published Online First: 2011/11/17]
41. Siddiqi S, Masud TI, Nishtar S, et al. Framework for assessing governance of the health system in developing countries: Gateway to good governance. *Health Policy* 2009;90(1):13-25. doi: http://dx.doi.org/10.1016/j.healthpol.2008.08.005
42. Walt G, Shiffman J, Schneider H, et al. 'Doing'Health Policy Analysis: Methodological and Conceptual Reflections and Challenges. *Health Policy and Planning* 2008;23(5):308-17.
43. Burke RM, Leon JS, Suchdev PS. Identification, prevention and treatment of iron deficiency during the first 1000 days. *Nutrients* 2014;6(10):4093-114.
44. Abshire TC. The anemia of inflammation. A common cause of childhood anemia. *Pediatr Clin North Am* 1996;43(3):623-37. doi: 10.1016/s0031-3955(05)70425-9 [published Online First: 1996/06/01]
45. WHO. World Health Organization. Serum ferritin concentrations for the assessment of iron status and iron deficiency in populations. 2011
46. Gerdes M, Garcia-Espana JF, Webb D, et al. Psychometric Properties of Two Developmental Screening Instruments for Hispanic Children in the Philadelphia Region. *Acad Pediatr* 2019;19(6):638-45. doi: 10.1016/j.acap.2018.10.002 [published Online First: 2018/10/14]

For peer review only

47. Clooney AG, Fouhy F, Sleator RD, et al. Comparing Apples and Oranges?: Next Generation Sequencing and Its Impact on Microbiome Analysis. *PLoS One* 2016;11(2):e0148028. doi: 10.1371/journal.pone.0148028 [published Online First: 2016/02/06]
48. Boers SA, Jansen R, Hays JP. Understanding and overcoming the pitfalls and biases of next-generation sequencing (NGS) methods for use in the routine clinical microbiological diagnostic laboratory. *European Journal of Clinical Microbiology & Infectious Diseases* 2019;38(6):1059-70. doi: 10.1007/s10096-019-03520-3
49. Dirección General de Salud Ambiental. Reglamento de la Calidad del Agua para Consumo Humano. . Lima, Perú: Ministerio de Salud - MINSA, 2011.
50. E.W. Rice RBB ADE, editors. . Standard Methods for the Examination of Water and Wastewater, 23rd Edition: American Public Health Association, American Water Works Association, Water Environment Federation 2017. 2017.
51. Haberzettl P, Lee J, Duggineni D, et al. Exposure to ambient air fine particulate matter prevents VEGF-induced mobilization of endothelial progenitor cells from the bone marrow. *Environ Health Perspect* 2012;120(6):848-56. doi: 10.1289/ehp.1104206 [published Online First: 2012/03/16]
52. van Eeden SF, Hogg JC. Systemic inflammatory response induced by particulate matter air pollution: the importance of bone-marrow stimulation. *J Toxicol Environ Health A* 2002;65(20):1597-613. doi: 10.1080/00984100290071685 [published Online First: 2002/10/25]
53. Morales-Ancajima VC, Tapia V, Vu BN, et al. Increased Outdoor PM2.5 Concentration Is Associated with Moderate/Severe Anemia in Children Aged 6–59 Months in Lima, Peru. *Journal of Environmental and Public Health* 2019;2019:8. doi: 10.1155/2019/6127845
54. Reyes-García M G-SPI, Espinoza-Barrientos, C.,. Tablas peruanas de composición de alimentos [Peruvian tables of food composition]. In: CENAN CNdAyN-, ed. Lima, Peru: Instituto Nacional de Salud - INS, 2017:146.
55. Reed M, Graves, A., Dandy, N., Posthumus, H., Hubacek, K., Morris, J., Prell, C., Stringer, L. C., & Quinn, C. H. Who's in and why? A typology of stakeholder analysis methods for natural resource management. *Journal of Environmental Management* 2009;90(5):1933-49. doi: https://doi.org/10.1016/j.jenvman.2009.01.001
56. Guest G, Namey E, McKenna K. How Many Focus Groups Are Enough? Building an Evidence Base for Nonprobability Sample Sizes. *Field Methods* 2016;29(1):3-22. doi: 10.1177/1525822X16639015
57. Hennink MM, Kaiser BN, Weber MB. What Influences Saturation? Estimating Sample Sizes in Focus Group Research. *Qualitative Health Research* 2019;29(10):1483-96. doi: 10.1177/1049732318821692
58. Blackman A, Fairey, T.,. The PhotoVoice Manual - A guide to designing and running participatory photography projects. UK: PhotoVoice, 2017:128.
59. Glaser BG, Strauss AL. Discovery of grounded theory: Strategies for qualitative research: Routledge 2017.

For peer review only

BMJ Open

Launching of the Anaemia Research PEruvian Cohort (ARPEC): A multicentre birth cohort project to explore the iron adaptive homeostasis, infant growth, and development in three Peruvian regions.

Journal:	*BMJ Open*
Manuscript ID	bmjopen-2020-045609.R1
Article Type:	Protocol
Date Submitted by the Author:	30-Mar-2021
Complete List of Authors:	Montag, Doreen; Queen Mary University of London, Centre for Global Public Health<br>Delgado, Carlos; Instituto Nacional de Salud del Nino; Universidad Nacional Mayor de San Marcos, Pediatric, Facultad de Medicina<br>Quispe, Consuelo; Universidad Nacional de San Cristóbal de Huamanga<br>Wareham, David; Queen Mary University of London, Blizard Institute<br>Gallo, Valentina; University of Groningen, Campus Fryslan<br>Sanchez-Choy, Jose; Universidad Nacional Intercultural de la Amazonia<br>Sanchez, Victor; Instituto Nacional de Salud del Nino<br>Anaya, Ruth; Universidad Nacional de San Cristobal de Huamanga<br>Flores, Elaine; Queen Mary University of London, Centre for Global Public Health<br>Roca, Lorena<br>Mamani, Victor ; Instituto Nacional de Salud del Niño<br>Rivera Medina, Juan; Instituto Nacional de Salud del Niño; Universidad Nacional Mayor de San Marcos, Facultad de Medicina<br>Velasquez, Pablo; Instituto Nacional de Salud del Niño; Instituto Nacional Materno Perinatal<br>Del Aguila, Carlos; Instituto Nacional de Salud del Niño; Universidad Nacional Federico Villarreal<br>Prendergast, Andrew ; Queen Mary University of London,<br>Palomino, Julio; Universidad Nacional Santiago Antúnez de Mayolo
**Primary Subject Heading**:	Nutrition and metabolism
Secondary Subject Heading:	Nutrition and metabolism, Paediatrics, Public health, Global health, Haematology (incl blood transfusion)
Keywords:	Anaemia < HAEMATOLOGY, Health policy < HEALTH SERVICES ADMINISTRATION & MANAGEMENT, MICROBIOLOGY, OBSTETRICS, PAEDIATRICS

I, the Submitting Author has the right to grant and does grant on behalf of all authors of the Work (as defined in the below author licence), an exclusive licence and/or a non-exclusive licence for contributions from authors who are: i) UK Crown employees; ii) where BMJ has agreed a CC-BY licence shall apply, and/or iii) in accordance with the terms applicable for US Federal Government officers or employees acting as part of their official duties; on a worldwide, perpetual, irrevocable, royalty-free basis to BMJ Publishing Group Ltd ("BMJ") its licensees and where the relevant Journal is co-owned by BMJ to the co-owners of the Journal, to publish the Work in this journal and any other BMJ products and to exploit all rights, as set out in our licence.

The Submitting Author accepts and understands that any supply made under these terms is made by BMJ to the Submitting Author unless you are acting as an employee on behalf of your employer or a postgraduate student of an affiliated institution which is paying any applicable article publishing charge ("APC") for Open Access articles. Where the Submitting Author wishes to make the Work available on an Open Access basis (and intends to pay the relevant APC), the terms of reuse of such Open Access shall be governed by a Creative Commons licence – details of these licences and which Creative Commons licence will apply to this Work are set out in our licence referred to above.

Other than as permitted in any relevant BMJ Author's Self Archiving Policies, I confirm this Work has not been accepted for publication elsewhere, is not being considered for publication elsewhere and does not duplicate material already published. I confirm all authors consent to publication of this Work and authorise the granting of this licence.

For peer review only

Launching of the Anaemia Research PEruvian Cohort (ARPEC): A multicentre birth cohort project to explore the iron adaptive homeostasis, infant growth, and development in three Peruvian regions.

Doreen Montag[1,9x], Carlos A. Delgado[2,3x], Consuelo Quispe[4], David W Wareham[6], Valentina Gallo[1,6,7,8], Jose Sanchez-Choy[9], Victor Sanchez[2], Ruth Anaya[5], Elaine C. Flores [1,7], Lorena Roca[5], Victor Mamani[2], Juan Rivera Medina[2,3,10], Pablo Velasquez[2,11], Carlos Del Aguila[2,12], Andrew Prendergast[5], Julio Palomino[13]

[1] Institute of Population Health Science, Queen Mary University of London, London, UK
[2] Instituto Nacional de Salud del Niño, Lima, Peru
[3] Department of Paediatrics, Faculty of Medicine. Universidad Nacional Mayor de San Marcos. Lima, Peru
[4] Universidad Nacional de San Cristóbal de Huamanga, Ayacucho, Peru
[5]Blizard Institute, Queen Mary University of London, London, UK
[6]University of Groningen, Netherlands
[7]Department of Population Health, London School of Hygiene and Tropical Medicine, London, UK
[8] Department of Epidemiology and Biostatistics, School of Public Health, Imperial College London, London, UK
[9] Universidad Nacional Intercultural de la Amazonía, Pucallpa, Peru
[10] Universidad Peruana Cayetano Heredia, Lima, Peru
[11]Instituto Nacional Materno Perinatal, Lima, Peru
[12]Universidad Nacional Federico Villarreal, Lima, Peru
[13]Universidad Nacional Santiago de Antuñez de Mayolo, Huaraz, Peru

[x] Equal contribution

Word count: Abstract: 317 Main Text: 5253 Figures: 2

Keywords: Anaemia, iron deficiency, Ecohealth, cohort, infant, Peru

Corresponding author
Dr Doreen Montag
Senior Lecturer of Global Health
Institute of Population Health Science, Queen Mary University of London, London, UK
Yvonne Carter Building, 58 Turner Street, London, E1 2AB
d.montag@qmul.ac.uk

For peer review only

Abstract

Background: Preventing infantile anaemia and ensuring optimal growth and development during early childhood, particularly in resource-constrained settings, represent an ongoing public health challenge. Current responses are aligned to treatment-based solutions, instead of determining the roles of its interrelated causes. This project aims to assess and understand the complex interplay of eco-bio-social-political factors that determine infantile anaemia to inform policy, research design and prevention practices.

Methods: This is a longitudinal birth cohort study including four components: 1. Biological, will assess known blood markers of iron homeostasis and anaemia and stool microbiota to identify and genetically analyse the participants' flora; 2. Ecological, will assess and map pollutants in air, water and soil and evaluate features of nutrition and perceived food security; 3. Social, which will use different qualitative research methodologies to explore key stakeholders and informants' perceptions related to nutritional, environmental and anaemia topics, participant observations and a participatory approach; and 4. A Political analysis, to identify and assess the impact of policies, guidelines and programs at all levels for infantile anaemia in the three regions. Finally, we will also explore the role of social determinants and demographic variables longitudinally for all study participants. This project aims to contribute to the evidence of the interrelated causal factors of infantile anaemia, addressing the complexity of influencing factors from diverse methodological angles. We will assess infantile anaemia in three regions of Peru, including newborns and their mothers as participants, from childbirth until their first year of age.

Ethics and Dissemination: Ethical approval was obtained from the Institutional Research Ethics Committee of the Instituto Nacional de Salud del Niño (Lima, Peru), CIEI-043-2019. An additional opinion has been granted by the Ethical Committee of Queen Mary University of London (London, United Kingdom). Dissemination across stakeholders is taking part as a continues part of the research process.

Summary

'Strengths and limitations of this study'

- There is scarce empirical evidence on the interrelated causes of infantile anaemia, which will be addressed in this project through an eco-bio-social-political approach
- Iron supplementation for infants seems not to be a solution in iron-deficiency anaemia.
- Prevention measurement effects remain unclear before six months of age.
- The project outcomes will inform policy, research design and prevention practices from a multidisciplinary evidence-based perspective.
- *The project might be limited by the current COVID-19 pandemic.*

For peer review only

Limitations: *The first great limitation to field research in the 2020s is the COVID-19 pandemic. Recruitment in the hospitals and visits during follow-up must be performed under extreme safety precaution against COVID-19 transmission. In a recent steering committee meeting, the argument of restricting our study population to carry it out was reinforced. The restriction may imply conducting the study just in two cities instead of three. If conditions continue to be adverse, it will also include a reduction in the sample size and procedures to be performed. However, in February 2021 the health personnel started vaccination in Peru and many of our researcher staff are related to health facilities. Consequently, we still hope to start recruiting in July 2021. The disparities among participating centres' laboratory facilities will also generate additional challenges to collect and ship the biological samples.*

Generalizability: *To obtain results replicable over space and time, it is mandatory to include any influential variant to achieve sufficient explanation to be true. The study uses an approach to comprehend the complex interplay of eco-bio-social-political factors on anemia in particular settings. While the approach as a methodology might show very useful to adapt to diverse settings the interplay of the factors is likely going to be different in diverse settings and might needs additional studies and might therefore not be generalizable.*

Introduction

Iron deficiency (ID) anaemia (IDA) is a pressing global health challenge, which impacts the auditory, visual, and neurological development of children[1-3]. By 2016, the worldwide prevalence of anaemia in children <5 years, reported in the WHO Global Health Observatory data repository was 41.7 % [38.1-45.9]. The major risk factors for IDA are food insecurity[4], malnutrition and poverty, all of which will be exacerbated under current and future climate conditions[5]. The Anthropocene is marked by global change influencing planetary processes, including biodiversity and land-use changes, impacting food security, soil and water health directly linked to IDA [6]. Characterising and solving the underlying complexity of the factors that interact and determine IDA for millions of children, living in different circumstances globally, will be crucial to adapt to future risks and to mitigate the burden of disease by developing a durable environmental governance focused on health[7-9].

In Peru, as in other low-and-middle-income countries (LMICs), ID and IDA represent a high burden of disease. Between the years 2000 and 2010, the prevalence was over 50% in children aged 6 to 35 months and by 2017 it remained around 40%[10]. Nationally in the same period, anaemia prevalence decreased by about 10%[11]. However, from 2012 to date, the prevalence rates of anaemia remain around 42.2%[10 12].

For peer review only

It is important to highlight, though, that treating a deficiency does not necessarily mean supplementing it. During early life, iron homeostasis controls are absent or limited, because the regulation mechanisms for this process develop in later infancy[13]. Prescription of iron supplementation during the first months of life, whether during breastfeeding or mixed breastfeeding, may interfere with nutrient absorption and may adversely affect the intestinal microbiome[14 15]. Also, there is sparse evidence that iron supplementation may correct neurodevelopmental deficits caused by a lack of iron in early childhood[16].

The eco-bio-socio-political framework in Peru

Political & economic factors

Over the last decades there has been a steady increase of the Gross domestic product (GDP) in Peru[17], with up to over 8% per year growth between the years 2000 and 2018[17] reflecting a commodity boom. The GDP, as an indicator of macro-economic development, should have had a direct impact on poverty reduction, decreased food insecurity indicators and improved health outcomes, leading to reduced IDA prevalence. However, this has not happened: high rates of infant anaemia still exist[18] suggesting a lack of a continuous systemic approach by the government, despite its importance on social and economic indicators for the Peruvian society.

Social factors

Food insecurity in Peru is rooted in Peruvian colonial history and imbalanced power relations. It is distributed unevenly in each ecological region, often merely addressed as a problem of poverty, which is poorly overseen by policies and interventions over the last 70 years[19]. Food insecurity and malnutrition are consequences of multidimensional factors related to broader social and economic inequalities. These, in turn, influence rural-urban migration patterns and configures budgetary restrictions for food and nutrition of women in their homes[20 21]. Gender relations within households' further influence power dynamics and subsequently has a direct impact on infant nutrition[22].

Biological factors

Biological factors encompass microbiota development and iron homeostasis in breastfeeding infants. During breastfeeding, there is a predominance of *Bifidobacterium* bacteria, that represent 60-90% of the total microbiota, due to the rich supply of oligosaccharides in breast milk. As complementary food gets introduced, these are successively displaced by *Firmicutes* and *Bacteroidetes*, capable of absorbing

For peer review only

complex polysaccharides derived from plant products appropriate for the stage of infant feeding and growth. Given the large concentration of lactoferrin (an iron-transporting protein) in breast milk, and its resistance to proteolysis, it can be detected in breastfeeding stools[23]. Optimal microbiota succession can be disrupted by environmental factors, such as exposure to unclean water and lack of sanitation, early introduction of complementary feeding during the period of exclusive breastfeeding, and the quality of the infant diet.

Ecological & environmental factors

Biodiversity loss, increased urbanisation and deteriorating ecosystems have a documented impact on gut microbiota and health[24 25]. Rook and Knight[26] argue that biodiversity of the soil, such as the green spaces that children mature in, plays a vital role in the bacteria induced immune modulation, and subsequently influences microbiota development and homeostasis. Anthropogenic climate change is altering biodiversity and iron and other mineral content of certain foods, further restricting access to food across vulnerable population groups and increasing food insecurity[27 28].

Water quality is influenced by biodiversity, deforestation, mining activities and general pollution. Water pollution, specifically, can lead to increased parasitic infections in infants, which in turn can compete for iron in the intestine[24 29]. Environmental enteropathy (EE), also called environmental enteric dysfunction[30], is a subclinical disorder of the small intestine which alters the structure and function of the gut among children living in settings with environmental contamination and high poverty rates. EE reduced gut barrier function, and intestinal malabsorption and all of these processes may contribute to IDA[31 32].

Rationale of the project

Given the complexity of the factors potentially increasing the risk of IDA, a comprehensive approach is needed to investigate and disentangle such complexity. This entails examining in detail the ecological, biological, social and political factors that would increase the risk for developing IDA in the first year of life among 330 children in Lima (Coast), Ayacucho (Andes) and Pucallpa (Amazon), who will be born to healthy mothers without anaemia during the year 2021. We are proposing an eco-bio-socio-political framework, which is influenced by the work edited by Charron[33] on 'Ecohealth research in practice', that emphasises the importance of participation of all stakeholders and an interdisciplinary methodology, gender equity and sustainability to increase potentially lasting policy impacts. With the base of an initial simple diagram (Figure 1) and the research outcomes, we plan to approach and

For peer review only

comprehend in-depth the complex interplay between the selected factors which will allow us to further develop this framework.

Figure 1. The Eco-bio-socio-political framework applied to iron deficiency anaemia in children in Peru - Source: Own elaboration (DM)

The Anemia Research Peruvian Cohort (ARPEC) project seeks to diminish the scientific and technical evidence-base gap, supporting the development of comprehensive and sustainable policy[7 20 21 34]. In Peru, as in most of Latin America, the majority of anaemia is related to ID, due to lower levels of iron in the diet and its coexistence with high rates of gastrointestinal issues that impairs iron absorption[35-37]. For example, in the Andean region of Ayacucho, children's dietary iron consumption is almost 25% below the recommended iron requirements by age[38]. Despite this, the interdisciplinary empirical literature on the tangled causes and long-term determinants of this highly prevalent challenge is almost non-existent.

The ARPEC project status is updated regularly on its webpage https://www.qmul.ac.uk/arpecproject/

Aim and objectives of the study

Understand the complexity of the eco-bio-socio-political factors that influence food insecurity of breastfeeding mothers and their new-borns in three Peruvian regions, altering mechanisms of adaptive iron homeostasis in infants within the Anthropocene.

Objectives

1. To evaluate the effects of infant exposure to ecological / environmental factors, including nutrition, water quality and air and soil pollution, and their potential influence on gut microbiome development and the mechanisms of adaptive iron homeostasis in infants.
2. To assess the contribution of biological factors, such as succession of the intestinal microbiota, to the incidence of IDA in breastfed infants during the first year of life. We will focus especially on those organisms with siderophore genes and a competitive role in intestinal iron absorption.
3. To identify social factors influencing dietary patterns and food insecurity and their association with ID or IDA in breastfed babies during the first year of life.

For peer review only

4. To analyse political factors, including current policies and success or failure of previous policies and lasting and sustainable policies, in addressing food insecurity and IDA in different Peruvian regions.
5. To develop a framework able to explain the complex interaction and interdependence of the eco-bio-social-political factors determining IDA in Peru.

Methods

Study design

ARPEC project is a prospective observational multi-centre birth cohort study that will be conducted in three regions within Peru: the coast (Lima), the Andes (Ayacucho) and the Amazon rainforest (Pucallpa) with integrated quantitative and qualitative methodologies. The recruitment of participants for the birth cohort study is expected to start in July 2021 and overall data selection to be finished by August 2022. Literature review, policy analysis and qualitative interviews with stakeholders have started earlier and are expected to be culminated after.

Setting

Peru is the third most megadiverse country in the world, home to 55 indigenous groups (51 in the Amazon and 4 in the Andes) with 48 different indigenous languages[39]. Diversity is reflected in the nutritional and food practices throughout the country. This study involves inhabitants from three different regions: 1. Lima, as the capital of Peru, is located at 101 meters above sea level in the central coast. Lima encompasses almost 9.5 million inhabitants (32.3% of the national population) in a desert environment. It has the lowest proportion nationally of children <5 years old (7.3%)[40]. By 2018, in the urban Lima, the prevalence rates of anaemia in children between 6-35 months was 35.9%[12] and in 2017, among women between 15 and 49 years old was 23.1%[10]. Stunting for Lima in 2019 was reported as 4.9%[12]. 2. Pucallpa, the capital of the regional department of Ucayali, located at 154 meters above sea level, in the central Peruvian Amazon rainforest. It has almost half a million inhabitants (1.7% of the national population). The proportion of children <5 years old in Ucayali is *11.8%*[40].Stunting among children <5 was 24.8%, one of the highest in the country. The prevalence of anaemia in children between 6-35 months was 56.4% in 2018[12], and the highest prevalence was observed in children between 0-8 months (62%)[11]. 3. Ayacucho, is located at 2,760 meters above sea level in the Andes of South-eastern Peru. This region was the most affected during the bloody internal conflict in Peruvian recent history

For peer review only

(1980-2000). The region has 616,176 inhabitants (2.1 % of the national population). The proportion of children <5 years old in Ayacucho is *8,8%*[40] In 2018, stunting prevalence was 18.5% in children <5 years of age, and anaemia affected 49.3% of children aged 6-35 months[12]. According to the 2017 Census (INEI 2017) Ayacucho women of childbearing age constitute almost 25.5% of the total population (in contrast to 26.7% nationwide). Almost 27% of women from Ayacucho were mothers[41].

Participant recruitment and selection criteria

The project will identify, select and recruit 330 pairs of mothers and new-borns who will be involved in the study. Potentially eligible participants will be identified from the official registries of full-term pregnancies at the recruitment centres in the three cities. *The recruitment centers are second level health establishments that organize the identification and monitoring of prenatal controls for pregnant women in their area of influence. Pregnancies with risk factors are referred to facilities with greater resolution capacity.* We will consider the following inclusion criteria: singleton births, delivered vaginally, with umbilical cord clamping after ≥60 seconds, new-borns weighing >2,500 grams, mother-baby hospitalised jointly and discharged before 72 hours of life, without diagnosis of perinatal infection, birth asphyxia or congenital malformation. Participant mothers aged +18 years, who are capable of understanding study procedures, providing informed consent, and full-time residents in the study area. The mothers will be assessed during the month before the childbirth, at the last prenatal check-up or retrospectively through review of clinical records. Exclusion criteria are: mothers with a diagnosis of anaemia on the last prenatal haemoglobin levels; who had delivery via caesarean section; received antibiotics during the last month of pregnancy and the first week of life (mothers or their new-borns). The selection criteria are based on a low level of complexity for primary or secondary level of care. Certainly, at a hospital in Lima there is a greater number of patients who end up in caesarean section (because they tend to be more difficult cases), but even in a national reference hospital, there are also enough cases of low level of complexity. It is expected that recruitment participants from a similar level of complexity might minimize the selection bias.

For peer review only

Sample size and power calculation

The study sampling will be purposeful. We plan to have a total sample of 330 mother/child pairs, recruiting 110 new-borns from each region, totalling 660 participants, and assuming a maximum

potential loss of follow-up of 10%. Our hypothesis is that selected ecological, biological, social, and political factors increase the risk of IDA in the first year of life among the new-born's participants from the three regions. To test this association, we will have a power of 90% at a significance level of 5%, to detect an average difference of 0.5 g/dL between two subgroups, and 75% power to detect an average difference of 0.4 g/dL. Both are important differences for public health decisions and are associated with relevant outcomes such as early childhood development. We estimate that the level of haemoglobin in infants will be similar to that of their mothers, as has been reported for the city of Pucallpa in Peru[42] (average 11.5 g/dL, DS 1.3). The sampling frame does not attempt to produce a sample which is proportional to the underlying population, rather a sample which maximises power to detect differences across regions (although not powered to formally test for interaction). Samples of similar size across the recruiting centres will maximise the ability of describing existing differences in one or more variables.

Patient and Public Involvement

The research question and methodology were developed and discussed by the Principal Investigators and Co-Investigators across the participating four institutions in Peru and the UK. The study does not involve patients. A discussion of the methodology with stakeholders at the beginning of the research project has informed the methodology and is informing the iterative process of the research process.

Study procedures

Current knowledge review

We will perform an in-depth review of the literature, combining a systematic search and review with a policy analysis. The systematic review will focus on current knowledge on ID and IDA in Peru and the impact of environmental factors on IDA. The policy analysis will be focused on stakeholders, content, context, and capacity analysis to understand past successes and the underlying reasons on why, where and what has not been successful tackling IDA in Peru[43 44]. Findings from reviews will inform the qualitative research component planning and methodology, comprising in-depth interviews (IDIs) and focus groups discussion (FGDs) sessions.

Assessments of anaemia

We will assess IDA through haemoglobin (Hb), ferritin and C-reactive protein (CRP) levels in blood samples at baseline (birth) and during follow-up study visits at 4, 8, and 12 months of age[45]. Mothers will be examined after childbirth and before hospital discharge. In addition, concentrations of serum hepcidin, soluble transferrin receptor (sTFR) and lead will also be evaluated. We will use WHO criteria

For peer review only

to adjust ferritin values for inflammation[46], as reflected by the CRP[47]. We will follow standard guidelines to draw laboratory samples from children, accompanied by their mothers or primary caregivers.

Basic anthropometric measurements

We will measure weight and length of children at baseline and at each follow-up visit, with a digital calibrated scale and an infant meter, respectively, following standard local procedures. Additionally, we will measure head circumference and abdominal circumference with a non-elastic metric tape. Weight, height and abdominal circumference of mothers will be taken at baseline and at the 12-month visit, following standard assessment procedures for adults. All measurements will be performed by trained fieldworkers, with experience in local health services. Child development will be assessed using a locally validated version of the Child Welfare Survey (SWYC)[48]. The SWYC is a free screening instrument and requires almost 15 minutes to complete. It includes questionnaires adapted to age milestones to assess three domains of child functioning: Developmental Domain, Emotional/Behavioral Domain and Family Context.

Metagenomic analysis of the microbiota

The child's stool microbiota will be analysed using whole genome sequencing techniques during the three follow-up visits at 4, 8 and 12 months of life. The samples will be collected, processed, stored, and shipped for analysis from Peru to the Blizard Institute, Queen Mary University of London (QMUL). We will also undertake phenotypic analysis, using selective culture media, to isolate viable bacterial pathogens in a central laboratory in Lima. Selective chromogenic bacterial culture media will be used for primary isolation and quantification of *Enterobacterales, Enterococci, Bifidobacterium, Lactobacilli, Bacteroides, Clostridia, Candida spp* and siderophore-producing isolates (CAS agar). Molecular analysis and next generation sequencing (NGS) of the faecal microbiome will be performed by metagenomic shotgun sequencing[49 50]. The DNA of the entire bacterial community will be extracted, fragmented and sequenced using Illumina HiSeq technology. Bioinformatic analysis of sequence data will be performed using the CosmosID Metagenomics Cloud application (https;//app.cosmosid/login) (Rockville, MA) to enable taxonomic profiling of bacteria, viruses, phages, fungi, protists, microbial resistome and virulome.

Water quality analysis

For peer review only

Sampling for water quality analysis will be conducted in two different seasons (dry and wet) and within comparative geographical reference points in each region (Pucallpa, Ayacucho, Lima), considering their relationship to other human activities, including mining, lack of sewage and other sanitary installations that have an impact on water quality. We will determine during fieldwork collection the pH, conductivity, turbidity, and percentage of dissolved oxygen in water samples. In addition, we will assess under laboratory conditions the levels of metals (such as iron, manganese, aluminium, copper, zinc, lead, mercury), hardness characteristics, presence of nutrients (such as phosphate, nitrate), and microbiologic characteristics (such as the presence and quantity of heterotrophic bacteria, total coliforms and thermotolerant coliforms) following the government's permitted parameters[51]. The assessments will follow the protocols of Standard Methods for Water and Wastewater[52] and laboratory guidelines of the World Health Organization.

Air quality analysis

We will evaluate air quality across the three regions using data from different monitors, satellite reports and field collection at pre-identified geographical landmarks during the study visits. Environmental exposure to air pollutants, fine particles equal to or less than 2.5 microns in diameter (PM2.5) and NO_2 may produce systemic inflammation and determine changes in the bone marrow and act as a direct risk factor for the development of anaemia[53 54]. A recent study conducted in Lima, Peru, determined a significant association between the increase in the level of PM 2.5 measured in the environment, with decreased haemoglobin values and an increase in the prevalence of moderate and severe anaemia in children under 5 years[55].

Geographic mapping

We will also explore the proposed relationship between environmental exposures and microbiota characteristics using the GIS georeferencing system. This will allow us to map the prevalence of anaemia through estimating the approximate distance to the child's home, the specific microbiota differences and the biodiversity level of the green areas surrounding the households[26].

Sociodemographic and nutritional evaluation procedures

We will assess socioeconomic and food practices variables among all participants. The nutritional instrument will be locally piloted and adapted for each region to provide information on the food groups consumed by the mother and infant beyond 4 months of age when new food is being introduced. This

For peer review only

will vary within regions, by seasonality factors and food access. We will also use locally *developed visual aids* materials to ease the description and characteristics of foods, drinks, and portion sizes of the food commonly consumed by the participants.

We acknowledge the potential importance of the participant's various socio-cultural backgrounds (Shipibo-Konibo, Quechua) and the different feeding practices during weaning, which will be explored in-depth through qualitative methodology. Our approach aims to be participatory since inception, following Eco health approaches[33] and has a high potential to develop participatory and lasting prevention policies. We will base our analysis on the known nutritional content of fruits and legumes nationally[56].

Qualitative Research Procedures

We will conduct in-depth interviews with participating mothers, regional and national key informants, and local stakeholders. We will aim to interview between 25-30 mothers (8-10 from each region) to reach saturation, selected by convenience. For the stakeholder interviews, one key stakeholder per institution will be selected. Given the high fluctuance of stakeholders in institutions and to comprehend the challenges of anaemia policies from a historic perspective a snowball method will be used to determine stakeholders and provide a comprehensive insight[57]. The purpose of interviews among stakeholders is to evaluate their perceptions of what are the causes for IDA and their opinion on the historical success or failure of policy interventions related to anaemia. 3-6 FGDs[58] will be conducted among mothers and stakeholders, separately, in each location to identify, discover and review important themes and provide meaning[59]. FGDs among key stakeholders in each region and on a national level will address two main areas: First, the perceived relationship between nutritional and environmental factors and the potential risks for the development or worsening of the iron deficiency anaemia among children. Secondly, they will address the policy level, which will be divided into two phases: 1. The experiences and perceptions of effective past and current policies and barriers to effective implementation of governmental interventions and lasting policies and 2. During the last part of the research period, after the presentation of the results, to discuss and inform potential policy implementation based on the research results and practical aspects of transforming policies into practice. Finally, we anticipate conducting a public engagement and participatory activity with a group of participating mothers from each region. This will use photography as a means of non-verbal expression,

For peer review only

allowing participants to identify, select, register and present everything that they consider important and related to the health of their new-borns and the risks related to the development of anaemia, following the PhotoVoice © method[60].

Operationalization of the variables

The list of variables and their characteristics will be included in the study protocol, instruments, manuals, and standard operations procedures.

Data analysis

We will conduct biological (microbiota/stool, water, blood analysis), socio-cultural (nutrition, environment, policy), geographic-mapping (GIS-location relationship between green spaces, biodiversity and gut microbiota), and mixed-methods research (nutrition/household survey, qualitative methodology).

Quantitative data analysis

We will review the distribution of any missing values and evaluating imputation options, normality for all quantitative variables and their correlations. We will conduct descriptive analyses of the distribution of all microbiome and lifestyle data between the different haemoglobin levels across all participants.

Surveyed quantitative variables will be categorised appropriately for each follow-up visit and we will estimate their changes over time. Biological samples will be analysed to evaluate its microbiota content, and we will identify the most frequent taxa for each sample. Repeated measurements will allow us to calculate the largest changes in microbiota composition during follow-up period. Principal Component Analysis will be used to identify the most distinctive taxa and their associated patterns of changes with the lowest haemoglobin levels at the end of the study period. These in turn will be analysed in association with other lifestyle variables to identify possible causal pathways.

A multivariate logistic regression model will be used to investigate the cross-sectional association between potential risk factors and haemoglobin levels in children, in each visit. Additionally, information on selected exposures will be collected four times to calculate cumulative exposure, which will be then evaluated against ID and IDA at the end of study period. Both cumulative exposures and their changes will be included in a final Cox model, including exposure variables at the starting point, and their relevant changes, to explore the risk associated with lower haemoglobin levels, during the first year of life.

For peer review only

Qualitative analysis

Policy analysis will be conducted, encompassing national and regional policies since 1979 to comprehend the development of pData obtained from participant observations, focus group sessions and in-depth interviews will be audio-recorded and transcribed *verbatim*. This will later be segmented, entered, coded, and analysed to identify a list of relevant themes and key concepts related to the topics of interest. Coding will continue throughout the research process, using the grounded theory approach to data analysis, and informing follow-up interviews and focus groups[61]. The analysis will compare the information by different subgroups of participants to identify and describe the similarities and divergences between key stakeholders, participants from different regions, age groups, and those living across different environmental and nutritional risks. The analysis will also include field notes, direct observation, and minutes of the researchers' meetings.

Ethics

Ethical approval was obtained from the Institutional Research Ethics Committee of the Instituto Nacional de Salud del Niño (Lima, Peru), CIEI-043-2019. A second opinion has also been granted by the Ethical Committee of Queen Mary University of London (London, United Kingdom). Further to that, institutional support was sought from local authorities, particularly indigenous government organisations, local hospitals and health centres.

Written informed consent will be sought from all participants prior to enrolment. Verbal consent and permanent explanation of research purposes will continue throughout the study.

To protect participant's confidentiality, all collected data will be linked to a unique identification code. Data will be recorded in paper form and electronically. The electronic information will be stored, backed-up, and secured by password protection in the RedCap© server. Paper forms will be stored in secure locked cabinets at the three Peruvian sites. All confidential information, including participant's contact details will also be stored in secured locations, accessible only to authorized research staff. Long-term data management will comply QMUL's research policy.

Any requests for biological samples will be processed following international standards. Importantly, only processed stool samples will be sent to the UK.

For peer review only

Plan for results dissemination

The study team has been following and will adhere to a detailed plan for dissemination of study outcomes, that will be used to publicise the research at a national and regional level. The outline dissemination methods will include periodic meetings with stakeholders, academic institutions that directly and indirectly collaborate with the study team, scientific publications in peer-reviewed journals, presentation in scientific events to allow engagement with the wider international academic society in the discussion of research results, creation and release of integral policy briefs for stakeholders at the regional and national level in Peru, teaching through research-based teachings in relevant participating institutions and modules and presence in social media and a dedicated study website and logo (figure 2).

Acknowledgment

We would like to thank stakeholders for their constructive feedback on our methodology and taking part in the iterative participatory process.

Author contribution

Doreen Montag – Principal investigator (MRC) at QMUL, London. Conceived study ideas, drafted protocol, and overall responsibility of study design and implementation. Drafted the current manuscript

Carlos A. Delgado – Director of the Research Circle (FONDECYT) Principal Investigator at INSN, Lima. Conceived study ideas, major contribution to protocol, and overall responsibility of study design and implementation in Peru.

Consuelo Quispe – Local Principal Investigador for UNSCH, Ayacucho. Responsible of conceiving and operationalising anthropometric data collection.

David Wareham – Co-investigator at QMUL. Responsible for conceiving and operationalising genetic analyses of the microbiome. Theoretical and logistic input on faeces sample collection and microbiota analyses

Jose Sanchez Choy – Co-investigator at UNIA. Manager of data collection in Pucallpa

Victor Sanchez – Co-investigator at INSN. Overall input on study design and biomedical background and measurements

Ruth Anaya – Co-investigador, UNSCH, Ayacucho. General input on operationalising anthropometric data collection.

Valentina Gallo – Co-investigator at QMUL, London. Overall advice on epidemiological study design and statistical analyses. General input on biomedical background and measurements for neurodevelopmental issues

Andrew Prendergast – Co-investigator at QMUL, London. Overall input on study design and implementation

For peer review only

Lorena Roca -Research Assistant at UNSCH, Ayacucho, general input on anthropometric data collection and study context in Ayacucho.

Elaine C. Flores – Study -coordinator, coordination of protocol writing, collaborating in this manuscript drafting.

Victor Mamani- Nutrition expert, intellectual contribution to nutritional component.

Juan Rivera Medina-Co-investigator at INSN, intellectual contribution to biological research component.

Pablo Velásquez, Co-investigator at INSN, intellectual contribution to biological research component.

Carlos Del Aguila- Co-investigator at INSN, intellectual contribution to biological research component.

Julio Palomino – Local principal investigator for UNIA, Pucallpa. Responsible of conceiving and operationalising environmental data collection. Theoretical and logistic input on faeces sample collection, microbiota, and environmental analyses.

Funding

This project is supported by the Medical Research Council – MRC grant number MR/S024654/1 and by the Fondo Nacional de Desarrollo Científico, Tecnológico y de Innovación Tecnológica – FONDECYT, grant number 033-2019. AJP is funded by the Wellcome Trust (108065/Z/15/Z).

Competing interest statement

None of the authors declare any competing interest

Figures

Figure 2

Arpec logo by Daddo Illustrations at www.daddoillustrations.com.

For peer review only

References

1. Algarín C, Peirano P, Garrido M, et al. Iron deficiency anemia in infancy: long-lasting effects on auditory and visual system functioning. *Pediatric research* 2003;53(2):217.
2. Allali S, Brousse V, Sacri AS, et al. Anemia in children: prevalence, causes, diagnostic work-up, and long-term consequences. *Expert Rev Hematol* 2017;10(11):1023-28. doi: 10.1080/17474086.2017.1354696 [published Online First: 2017/10/13]
3. Lozoff B, Beard J, Connor J, et al. Long-lasting neural and behavioral effects of iron deficiency in infancy. *Nutr Rev* 2006;64(5 Pt 2):S34-43. [published Online First: 2006/06/15]
4. FAO. State of Food Insecurity in the World: Meeting the 2015 International Hunger Targets: Taking Stock of uneven Progress. Rom: FAO, 2015.
5. Crutzen PJ. Geology of Mankind. In: Crutzen PJ, Brauch HG, eds. Paul J Crutzen: A Pioneer on Atmospheric Chemistry and Climate Change in the Anthropocene: Springer 2016:211-15.
6. Rockström J, Steffen W, Noone K, et al. Planetary Boundaries: Exploring the Safe Operating Space for Humanity. *Ecology and Society* 2009;14(2):32.
7. Montag D, Kuch U, Rodriguez L, et al. Overview of the Panel on Biodiversity and Health under Climate Change In: Diversity CoB, ed. The Lima Declaration on Biodiversity

   Climate Change: Contributions from Science to Policy for Sustainable Development: Convention of Biological Diversity 2017:91-108.
8. Watts N, Adger WN, Agnolucci P, et al. Health and Climate Change: Policy Responses to Protect Public Health. *The Lancet* 2015;386(10006):1861-914.
9. Whitmee S, Haines A, Beyrer C, et al. Safeguarding Human Health in the Anthropocene Epoch: Report of the Rockefeller Foundation–Lancet Commission on Planetary Health. *The Lancet* 2015;386(10007):1973-2028. doi: 10.1016/S0140-6736(15)60901-1
10. INEI. Instituto Nacional de Estadística e Informática. Encuesta Demográfica y de Salud Familiar - ENDES 2017. Lima: Instituto Nacional de Estadística e Informática, 2017.
11. INEI. Instituto Nacional de Estadística e Informática. Encuesta Demográfica y de Salud Familiar - ENDES 2016. Lima: Instituto Nacional de Estadística e Informática, 2016.
12. INEI. Instituto Nacional de Estadística e Informática. Encuesta Demográfica y de Salud Familiar - ENDES 2019 (resultados preliminares). Lima: Instituto Nacional de Estadística e Informática, 2019.
13. Lonnerdal B. Development of iron homeostasis in infants and young children. *Am J Clin Nutr* 2017;106(Suppl 6):1575s-80s. doi: 10.3945/ajcn.117.155820 [published Online First: 2017/10/27]
14. Dewey KG, Domellöf M, Cohen RJ, et al. Iron supplementation affects growth and morbidity of breast-fed infants: results of a randomized trial in Sweden and Honduras. *The Journal of nutrition* 2002;132(11):3249-55.
15. Paganini D, Zimmermann MB. The effects of iron fortification and supplementation on the gut microbiome and diarrhea in infants and children: a review. *Am J Clin Nutr* 2017;106(Suppl 6):1688s-93s. doi: 10.3945/ajcn.117.156067 [published Online First: 2017/10/27]
16. Cerami C. Iron Nutriture of the Fetus, Neonate, Infant, and Child. *Ann Nutr Metab* 2017;71 Suppl 3:8-14. doi: 10.1159/000481447 [published Online First: 2017/12/22]
17. The World Bank Group. Peru 2019 [cited 2019. Available from: https://data.worldbank.org/country/peru accessed February 27th, 2019 2019.
18. Alcázar L. The economic impact of anaemia in Peru: Lima 2013.
19. Smith LC, El Obeid AE, Jensen HH. The geography and causes of food insecurity in developing countries. *Agricultural economics* 2000;22(2):199-215.
20. Leatherman T, Jernigan K. The Reproduction of Poverty and Poor Health in the Production of Health Disparities in Southern Peru. *Annals of Anthropological Practice* 2014;38(2):284-99.
21. Oths KS, Smith HN, Stein MJ, et al. A decade of rapid change: Biocultural influences on child growth in highland Peru. *American Journal of Human Biology* 2018;30(2):e23072.
22. Serrano CA, Harris PR. [Development of intestinal microbiome in children. Impact on health and disease]. *Rev Chil Pediatr* 2016;87(3):151-3. doi: 10.1016/j.rchipe.2016.04.002 [published Online First: 2016/05/12]
23. Lonnerdal B, Georgieff MK, Hernell O. Developmental physiology of iron absorption, homeostasis, and metabolism in the healthy term infant. *The Journal of Pediatrics* 2015;167(4):S8-S14.
24. WHO/CBD. Connecting Global Priorities: Biodiversity and Human Health. A State of Knowledge Review.: World Health Organization and Secretariat of the Convention on Biological Diversity 2015.

For peer review only

25. Rook GA. Regulation of the Immune System by Biodiversity from the Natural Environment: An Ecosystem Service Essential to Health. *Proceedings of the National Academy of Sciences* 2013;110(46):18360-67.
26. Rook GA, Knight R. Environmental Microbial Diversity and Noncommunicable Diseases. In: WHO/CBD, ed. Connecting Global Priorities: Biodiversity and Human Health A State of Knowledge Review. Geneva: World Health Organization and Secretariat of the Convention on Biological Diversity 2015:151-64.
27. Torp Austvoll C, Gallo V, Montag D. Health impact of the Anthropocene: the complex relationship between gut microbiota, epigenetics, and human health, using obesity as an example. *Glob Health Epidemiol Genom* 2020;5:e2. doi: 10.1017/gheg.2020.2 [published Online First: 2020/05/05]
28. Phalkey RK, Aranda-Jan C, Marx S, et al. Systematic review of current efforts to quantify the impacts of climate change on undernutrition. *Proceedings of the National Academy of Sciences* 2015;112(33):E4522-E29.
29. Hudak L, Jaraisy A, Haj S, et al. An updated systematic review and meta-analysis on the association between Helicobacter pylori infection and iron deficiency anemia. *Helicobacter* 2017;22(1) doi: 10.1111/hel.12330 [published Online First: 2016/07/14]
30. Weinhouse C, Ortiz EJ, Berky AJ, et al. Hair Mercury Level is Associated with Anemia and Micronutrient Status in Children Living Near Artisanal and Small-Scale Gold Mining in the Peruvian Amazon. *Am J Trop Med Hyg* 2017;97(6):1886-97. doi: 10.4269/ajtmh.17-0269 [published Online First: 2017/10/11]
31. Owino V, Ahmed T, Freemark M, et al. Environmental Enteric Dysfunction and Growth Failure/Stunting in Global Child Health. *Pediatrics* 2016;138(6):e20160641. doi: 10.1542/peds.2016-0641 [published Online First: 2016/12/13]
32. Watanabe K, Petri WA, Jr. Environmental Enteropathy: Elusive but Significant Subclinical Abnormalities in Developing Countries. *EBioMedicine* 2016;10:25-32. doi: 10.1016/j.ebiom.2016.07.030 [published Online First: 2016/08/09]
33. Charron DF. Ecohealth Research in Practice. Ottawa, Cairo, Dakar, Montevideo, Nairobi, New Delhi, Singapore: Springer, IDRC, CRDI 2012.
34. Acosta AM, Haddad L. The politics of success in the fight against malnutrition in Peru. *Food Policy* 2014;44:26-35.
35. Mujica-Coopman MF, Brito A, Lopez de Romana D, et al. Prevalence of Anemia in Latin America and the Caribbean. *Food Nutr Bull* 2015;36(2 Suppl):S119-28. doi: 10.1177/0379572115585775 [published Online First: 2015/07/01]
36. Prendergast A, Kelly P. Enteropathies in the developing world: neglected effects on global health. *Am J Trop Med Hyg* 2012;86(5):756-63. doi: 10.4269/ajtmh.2012.11-0743 [published Online First: 2012/05/05]
37. Bartelt LA, Bolick DT, Guerrant RL. Disentangling Microbial Mediators of Malnutrition: Modeling Environmental Enteric Dysfunction. *Cell Mol Gastroenterol Hepatol* 2019;7(3):692-707. doi: 10.1016/j.jcmgh.2018.12.006 [published Online First: 2019/01/11]
38. Fundación Accion Contra el Hambre. Aproximación al Consumo de Alimentos y Prácticas de Alimentación y Cuidado Infantil en Niños y Niñas de 6 a 23 meses de Edad - Realizado en familias de la provincia de Vilcashuamán, región Ayacucho. . In: Rivera HTF, ed. 1 ed. Lima: Acción contra el hambre

, 2012:1-74.
39. INEI. Instituto Nacional de Estadística e Informática 2009 [
40. INEI. Censo nacional. Lima: Instituto Nacional de Estadistico e Informatica, 2017.
41. INEI. Lima, Peru: Instituto Nacional de Estadistica e Informatica, 2017.
42. Gonzales GF, Tapia V, Gasco M, et al. [Maternal hemoglobin in Peru: regional differences and its association with adverse perinatal outcomes]. *Rev Peru Med Exp Salud Publica* 2011;28(3):484-91. [published Online First: 2011/11/17]
43. Siddiqi S, Masud TI, Nishtar S, et al. Framework for assessing governance of the health system in developing countries: Gateway to good governance. *Health Policy* 2009;90(1):13-25. doi: http://dx.doi.org/10.1016/j.healthpol.2008.08.005
44. Walt G, Shiffman J, Schneider H, et al. 'Doing'Health Policy Analysis: Methodological and Conceptual Reflections and Challenges. *Health Policy and Planning* 2008;23(5):308-17.
45. Burke RM, Leon JS, Suchdev PS. Identification, prevention and treatment of iron deficiency during the first 1000 days. *Nutrients* 2014;6(10):4093-114.
46. Abshire TC. The anemia of inflammation. A common cause of childhood anemia. *Pediatr Clin North Am* 1996;43(3):623-37. doi: 10.1016/s0031-3955(05)70425-9 [published Online First: 1996/06/01]
47. WHO. World Health Organization. Serum ferritin concentrations for the assessment of iron status and iron deficiency in populations. 2011

For peer review only

48. Gerdes M, Garcia-Espana JF, Webb D, et al. Psychometric Properties of Two Developmental Screening Instruments for Hispanic Children in the Philadelphia Region. *Acad Pediatr* 2019;19(6):638-45. doi: 10.1016/j.acap.2018.10.002 [published Online First: 2018/10/14]
49. Clooney AG, Fouhy F, Sleator RD, et al. Comparing Apples and Oranges?: Next Generation Sequencing and Its Impact on Microbiome Analysis. *PLoS One* 2016;11(2):e0148028. doi: 10.1371/journal.pone.0148028 [published Online First: 2016/02/06]
50. Boers SA, Jansen R, Hays JP. Understanding and overcoming the pitfalls and biases of next-generation sequencing (NGS) methods for use in the routine clinical microbiological diagnostic laboratory. *European Journal of Clinical Microbiology & Infectious Diseases* 2019;38(6):1059-70. doi: 10.1007/s10096-019-03520-3
51. Dirección General de Salud Ambiental. Reglamento de la Calidad del Agua para Consumo Humano. . Lima, Perú: Ministerio de Salud - MINSA, 2011.
52. E.W. Rice RBB ADE, editors. . Standard Methods for the Examination of Water and Wastewater, 23rd Edition: American Public Health Association, American Water Works Association, Water Environment Federation 2017. 2017.
53. Haberzettl P, Lee J, Duggineni D, et al. Exposure to ambient air fine particulate matter prevents VEGF-induced mobilization of endothelial progenitor cells from the bone marrow. *Environ Health Perspect* 2012;120(6):848-56. doi: 10.1289/ehp.1104206 [published Online First: 2012/03/16]
54. van Eeden SF, Hogg JC. Systemic inflammatory response induced by particulate matter air pollution: the importance of bone-marrow stimulation. *J Toxicol Environ Health A* 2002;65(20):1597-613. doi: 10.1080/00984100290071685 [published Online First: 2002/10/25]
55. Morales-Ancajima VC, Tapia V, Vu BN, et al. Increased Outdoor PM2.5 Concentration Is Associated with Moderate/Severe Anemia in Children Aged 6–59 Months in Lima, Peru. *Journal of Environmental and Public Health* 2019;2019:8. doi: 10.1155/2019/6127845
56. Reyes-García M G-SPI, Espinoza-Barrientos, C.,. Tablas peruanas de composición de alimentos [Peruvian tables of food composition]. In: CENAN CNdAyN-, ed. Lima, Peru: Instituto Nacional de Salud - INS, 2017:146.
57. Reed M, Graves, A., Dandy, N., Posthumus, H., Hubacek, K., Morris, J., Prell, C., Stringer, L. C., & Quinn, C. H. Who's in and why? A typology of stakeholder analysis methods for natural resource management. *Journal of Environmental Management* 2009;90(5):1933-49. doi: https://doi.org/10.1016/j.jenvman.2009.01.001
58. Guest G, Namey E, McKenna K. How Many Focus Groups Are Enough? Building an Evidence Base for Nonprobability Sample Sizes. *Field Methods* 2016;29(1):3-22. doi: 10.1177/1525822X16639015
59. Hennink MM, Kaiser BN, Weber MB. What Influences Saturation? Estimating Sample Sizes in Focus Group Research. *Qualitative Health Research* 2019;29(10):1483-96. doi: 10.1177/1049732318821692
60. Blackman A, Fairey, T.,. The PhotoVoice Manual - A guide to designing and running participatory photography projects. UK: PhotoVoice, 2017:128.
61. Glaser BG, Strauss AL. Discovery of grounded theory: Strategies for qualitative research: Routledge 2017.

For peer review only

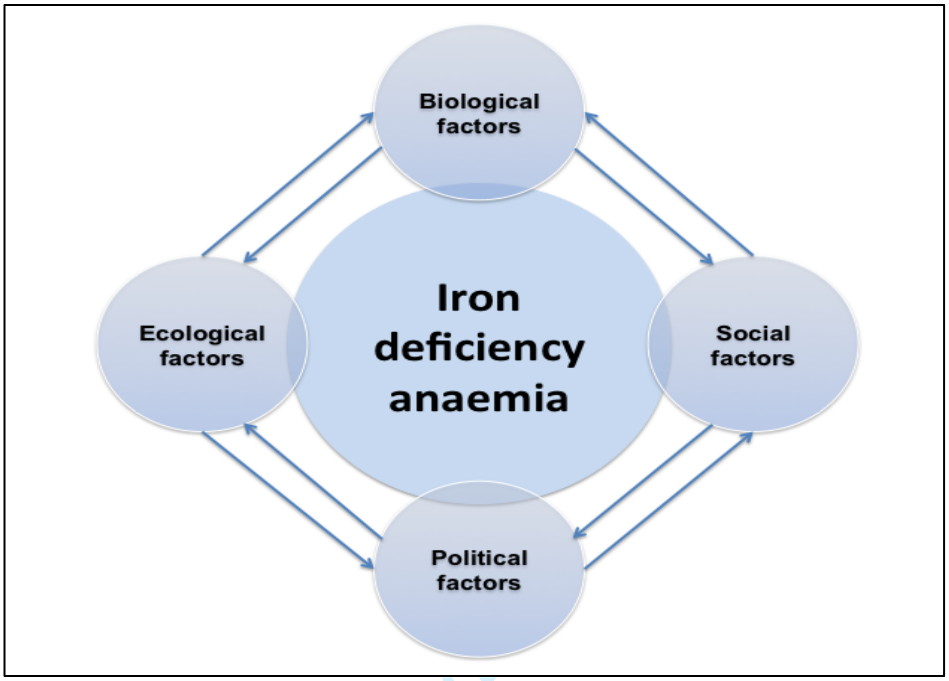

Figure 1. The Eco-bio-socio-political framework applied to iron deficiency anaemia in children in Peru - Source: Own elaboration (DM)

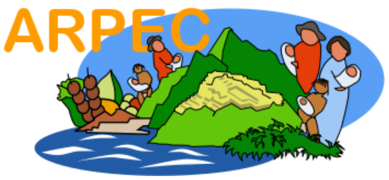

Figure 2: Arpec logo by Daddo Illustrations at www.daddoillustrations.com.

For peer review only
